# Living the Masterclass: Multiple Sclerosis Patient Journeys and the Reality of Participatory Medicine

**DOI:** 10.2196/94593

**Published:** 2026-10-08

**Authors:** Mary Hennings, Meredith O'Brien, Johanna Hising DiFabio, Jennifer Southard, Daniel van Leeuwen

**Affiliations:** 1 Society for Participatory Medicine, PO Box 610231, Newton Highlands, MA, 02461, United States, 1 9783942413; 2 Northeastern University, Boston, MA, United States; 3 Massachusetts Institute of Technology, Cambridge, MA, United States; 4 Adelphi University, Garden City, NY, United States; 5 Glenville State College, Arlington, MA, United States

**Keywords:** participatory medicine, multiple sclerosis, patient journey, patient preferences, patient empowerment

## Abstract

This reflective article, written collaboratively by 4 people living with multiple sclerosis (MS)—Johanna Hising DiFabio, Meredith O’Brien, Jennifer Southard, and Danny van Leeuwen—with Mary Hennings of the Society for Participatory Medicine, distills insights shared at a March 2025 event cohosted with Northeastern University’s Bouvé College of Health Sciences. The panelists speak as experts on their own lived experience, tracing the arc from diagnosis to what they call “optimal living” with a complex, unpredictable disease. The piece begins with the diagnostic journey itself, describing a “double trauma”: the anguish of unexplained physical decline before diagnosis, followed by the weight of confronting a confirmed chronic illness. The authors argue that how clinicians deliver and support a diagnosis determines whether patients end up isolated or drawn into genuine partnership with their care team. A central theme is the search for the right clinician fit—not just competence, but shared communication style and mutual respect for the patient’s expertise in their own life. The authors describe practices that support this, such as setting the visit agenda in advance through patient portals, and stress that patient engagement styles vary: some patients want deep involvement in research, while others prefer to delegate, but all need a clinician who genuinely listens. The article expands the notion of a care team beyond medical clinicians, describing three layers: the medical core, a complementary team (bodywork, movement, nutrition specialists), and a personal network of family and friends providing emotional support. It also names systemic frictions, like patients having to relay information among clinical specialists who don’t communicate with each other. A recurring message is resisting being reduced to the disease. The authors describe both the comfort and sting of comments like being told they “don’t look sick.” They also share personal strategies for preserving identity and quality of life, from adapting a musical instrument to accommodate physical limitations, to writing and publishing patient narratives, to using organizational resources like the National MS Society to navigate research and advocacy. The article closes with practical counsel for newly diagnosed patients: stay engaged, insist on true clinical partnership, build a broad support ecosystem, and protect the personal choices that give life meaning. It affirms the core principle of participatory medicine—that patients and clinicians grow together, with the patient retaining ownership of their own body and life.

## Introduction

This collective reflection explores what it means to evolve from a vulnerable person receiving a life-altering diagnosis into an empowered, equipped, engaged, and enabled e-patient who orchestrates our own care. It summarizes our observations about that journey. Other patients may find these insights helpful as they travel their own paths from diagnosis to optimal living with a complex, impactful disease.

## The Trauma of the Threshold: From Presentation to Diagnosis

The road to a multiple sclerosis (MS) diagnosis is seldom a straight line; it can be a confusing, agonizing maze. Because the disease presents in shifting, unpredictable ways, some of us were diagnosed quickly, while others spent years being dismissed, misdiagnosed, or told our worsening physical symptoms were psychosomatic [1].

This journey carries a double layer of trauma:

- The trauma of the unknown: living with a body that is failing without knowing why, while fighting to have our reality validated by clinicians.
- The trauma of the known: the heavy anxiety of the final diagnosis, where the fear of the unknown transforms into the stark reality of facing a chronic, progressive disease.

The diagnostic experience permanently shapes how a person interacts with clinicians. One of us was actively advised *not* to research their own condition—an instruction to remain passive. Another found immediate transformation by plugging into patient networks through the National MS Society [2]. The lesson here is foundational: how a diagnosis is delivered and supported dictates whether a patient is pushed into isolation or invited into partnership.

## Beyond Competence: Finding the Right Clinician Partner

We learned that clinical competence is the baseline; physician fit is not automatic. True participatory medicine requires compatibility in communication, management philosophy, and a mutual valuation of expertise [3].

Many of us walked away from our first neurologists. For some, a metrics-driven approach built around managing MS strictly “by the numbers” can ignore the priorities of the person living with the disease. We looked instead for clinicians who viewed us as collaborators in care.

In a successful partnership, communication flows both ways before anyone steps into the exam room:

- Setting the agenda: sharing current health status, concerns, and questions in advance through patient portals sets an agenda that increases the likelihood that clinical visits are efficient and effective.
- Varying styles of engagement: participatory medicine is not one-size-fits-all. Some of us want to dive deep into the latest clinical trials and research findings, while others prefer to delegate that synthesis to our doctor or care partners. The constant requirement, however, is that the clinician listens and acts upon what they hear from us.

Those of us who found alignment with clinicians in our team view our primary clinician as the medical quarterback. We know teamwork is a two-way street. As patients, we learn to navigate our clinicians’ unique styles, quirks, and constraints to build mutually resilient empathies [4,5].

## Cultivating the Wider Team

Active management of a complex condition like MS requires looking far beyond the conventional clinic walls. While the medical team remains essential, a rich, full life demands a broader ecosystem [2]. We think of our care infrastructure in three distinct layers (Textbox 1).

Textbox 1. **The three elements of care infrastructure.**

The medical core: neurologists, specialists, and primary care providers who manage disease modification and clinical metrics.

The complementary team: chiropractors, massage therapists, acupuncturists, yoga instructors, and dietary therapists who contribute to day-to-day comfort.

The personal network: family, friends, neighbors, and care partners who understand the context of our lives and provide emotional scaffolding.

Navigating this ecosystem reveals systemic failures. For example, patients are routinely forced to act as the “human fax machine,” serving as the communication glue between clinicians who do not talk to one another. The diversity of this ecosystem reveals internal tensions. Our family care partners may worry about progression, while we are consumed by the weight of the unintended consequences of today’s treatment. Naming these tensions openly helps the team move forward together.

## The Reality of Living Outside the Exam Room

Medical care happens in brief, intermittent flashes; the reality of a disease happens outside the exam room [6]. Life is punctuated by logistical mountains that healthy people navigate automatically. For instance, engineering the journey between an airport entrance and an airplane seat or managing the complex intersection of disease therapy with pregnancy or menopause are not straightforward for those of us with MS.

For us, thriving means refusing to allow the disease to consume our identity. We are not MS. Some people, when learning we have MS, say “But you look so good, I would never know you have MS.” Sometimes, we take the comment as a compliment: “Good, we’re not MS.” Other times, it feels like a minimization of the invisible, exhausting effort required to appear well. Many people offer fixes: “Have you tried drug x or diet y?” So, we don’t want to be defined solely by our diagnosis, but we need our burden to be seen, heard, and acknowledged without someone immediately trying to “fix” it.

Participatory medicine shines bright when clinicians partner with us to solve problems while preserving our humanity. For instance, one of us successfully negotiated a complex medication adjustment with her neurologist specifically so she could continue to wear high heels. To the clinician, it was a question of drug levels; to the patient, it was a nonnegotiable anchor of self-worth and identity. When a lifestyle choice carries that much meaning, it is worth innovating around.

## Tailored Strategies for a Rich Life

To move the needle from surviving to thriving, we have each had to innovate, leaning heavily on trusted resources and creative problem-solving:

- Danny’s strategy: As a dedicated saxophonist, holding a 12-pound instrument became impossible due to MS. Double vision hampered reading scores. However, realizing that breath work [7] was vital for respiratory health, a collaborative effort with a music teacher, an optometrist, and a German manufacturing company resulted in a custom-scaled instrument stand and an iPad-based music reader to mitigate the double vision. Working with nonclinical specialists can help preserve the things that feed the soul.
- Meredith’s strategy: As a writer and college teacher, purpose was found in translating lived experience into advocacy. Publishing *Uncomfortably Numb 2* [8], an anthology of patient stories, created a direct pipeline of practical wisdom to support the newly diagnosed, proving that patient stories enrich both the reader and the storyteller.
- Johanna & Jennifer’s strategy: Using digital anchors like the National MS Society website [2] provides an essential roadmap for everything from policy advocacy to self-care. For Jennifer, it answered the critical questions she didn’t yet know to ask as a newly diagnosed patient; today, it allows her to co-navigate emerging research side by side with her neurologist.

## Conclusion

Our collective journey offers a guide for newcomers navigating chronic, long-term illness. Our counsel: stay fiercely engaged, demand clinicians who operate as true partners, build an expansive care team, and aggressively protect the lifestyle choices that give your life meaning.

Keep in mind that the ultimate truth of the participatory movement: it is your body, it is your life, and as partners in health care, we grow together.

## Abbreviations

MS: multiple sclerosis
